# Supplementary figures and images for: PTH regulates osteogenesis and suppresses adipogenesis through Zfp467 in a feed-forward, PTH1R-cyclic AMP-dependent manner
Source: eLife. 2023 Apr 26;12:e83345. doi: 10.7554/eLife.83345 (PMC10171860; doi:10.7554/eLife.83345)

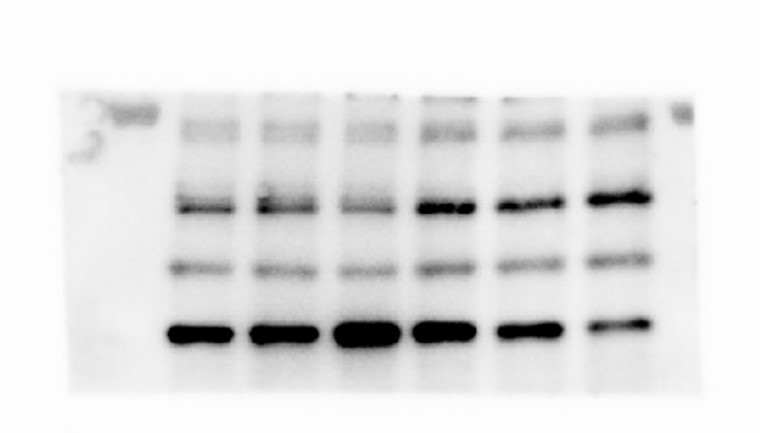

Supplement: Figure 3—source data 2. [file elife-83345-fig3-data2.zip › Source data 2 for Figure 3.jpg]

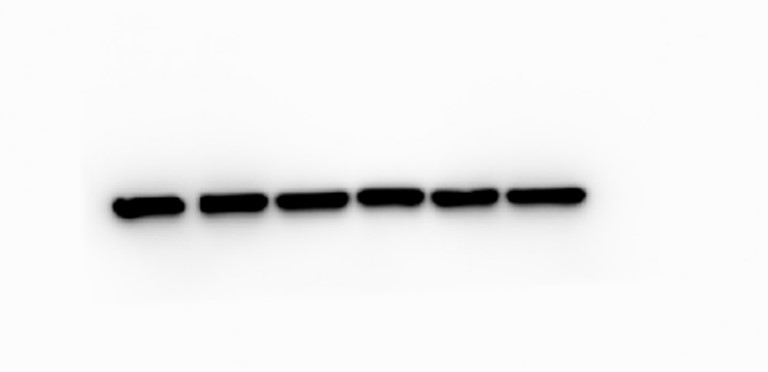

Supplement: Figure 3—source data 3. [file elife-83345-fig3-data3.zip › Source data 3 for Figure 3.jpg]

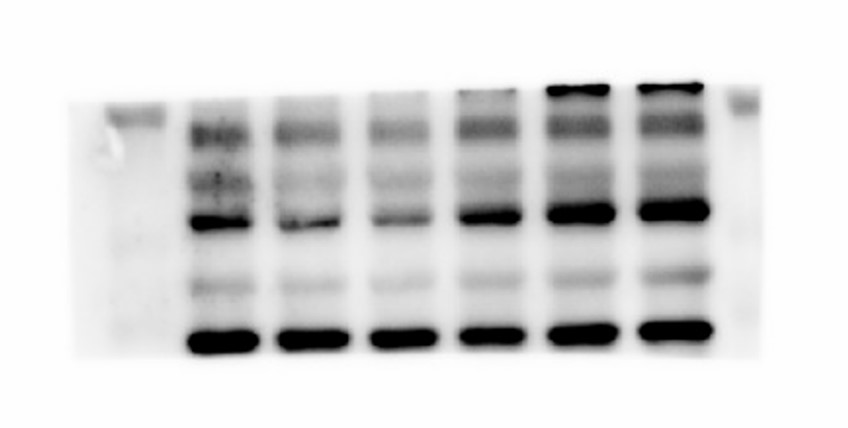

Supplement: Figure 3—source data 4. [file elife-83345-fig3-data4.zip › Source data 4 for Figure 3.jpg]

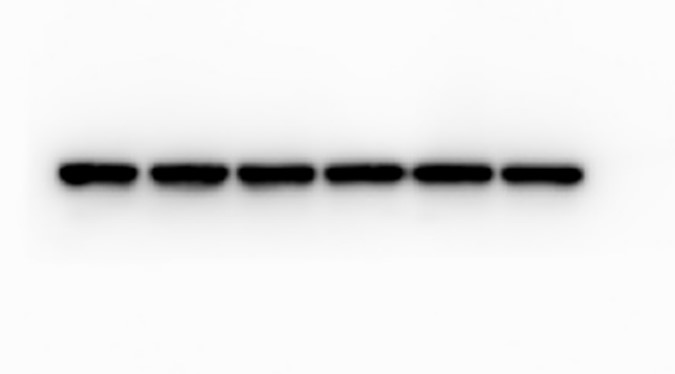

Supplement: Figure 3—source data 5. [file elife-83345-fig3-data5.zip › Source data 5 for Figure 3.jpg]

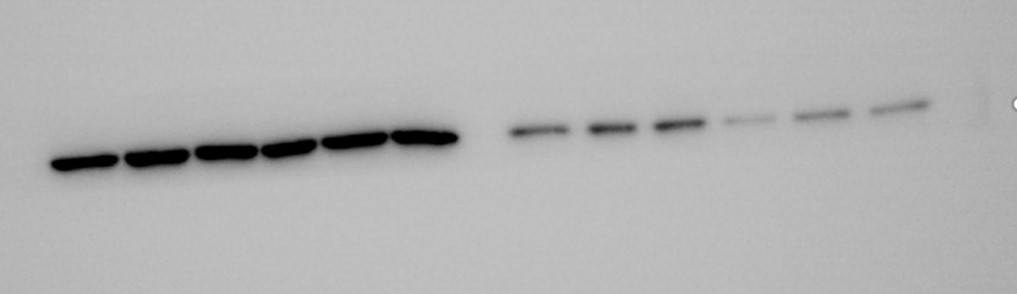

Supplement: Figure 4—source data 2. [file elife-83345-fig4-data2.zip › Source data 2 for Figure 4.jpg]

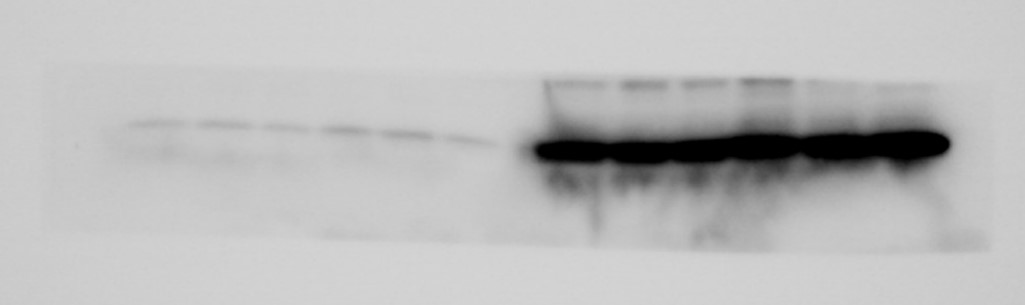

Supplement: Figure 4—source data 3. [file elife-83345-fig4-data3.zip › Source data 3 for Figure 4.jpg]

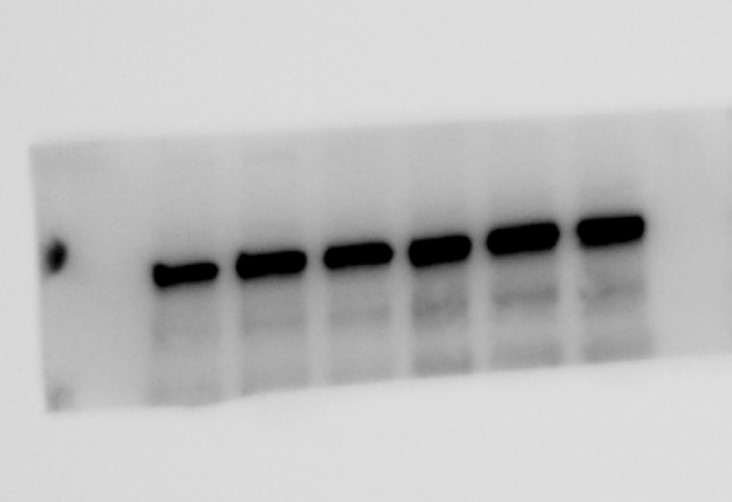

Supplement: Figure 4—source data 4. [file elife-83345-fig4-data4.zip › Source data 4 for Figure 4.jpg]

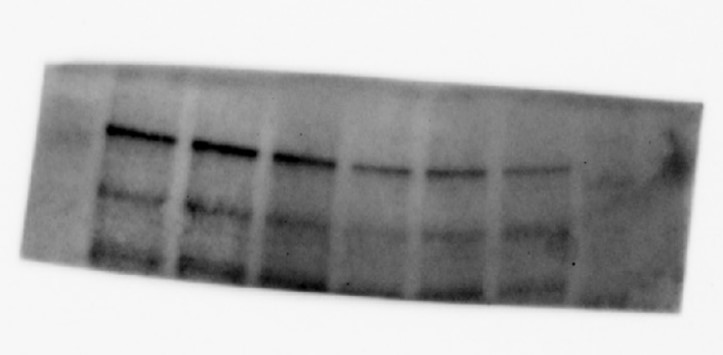

Supplement: Figure 4—source data 5. [file elife-83345-fig4-data5.zip › Source data 5 for Figure 4.jpg]

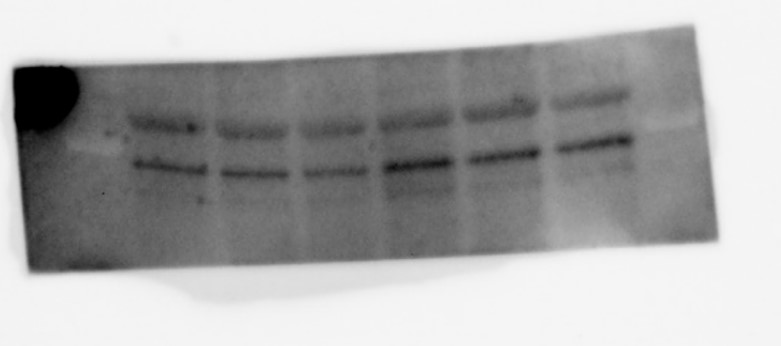

Supplement: Figure 4—source data 6. [file elife-83345-fig4-data6.zip › Source data 6 for Figure 4.jpg]

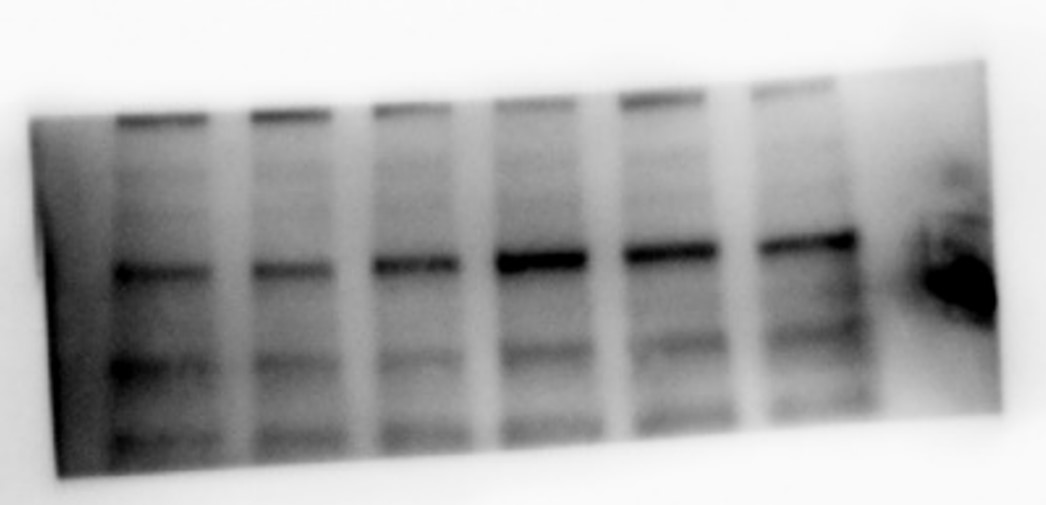

Supplement: Figure 4—source data 7. [file elife-83345-fig4-data7.zip › Source data 7 for Figure 4.jpg]

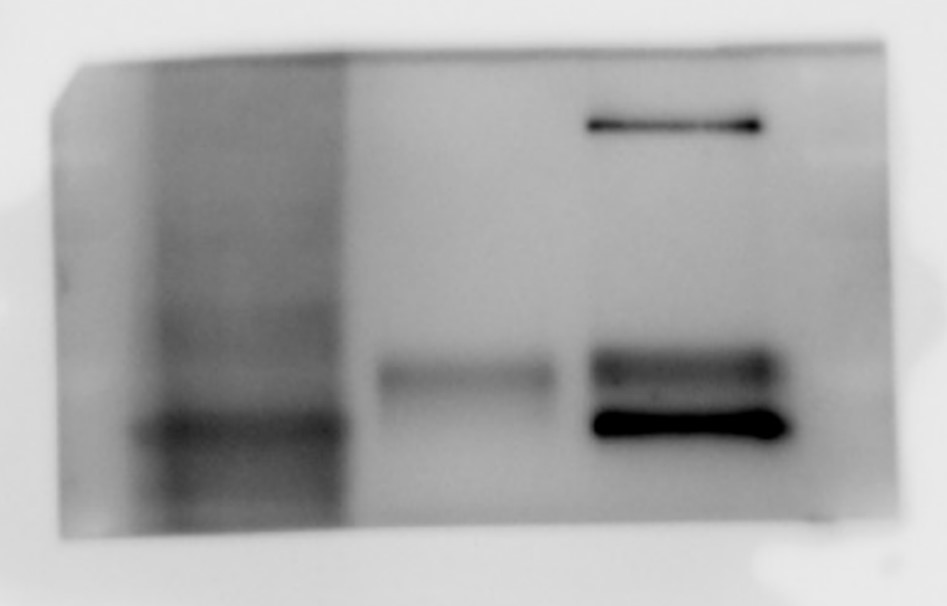

Supplement: Figure 5—source data 2. [file elife-83345-fig5-data2.zip › Source data 2 for Figure 5.jpg]

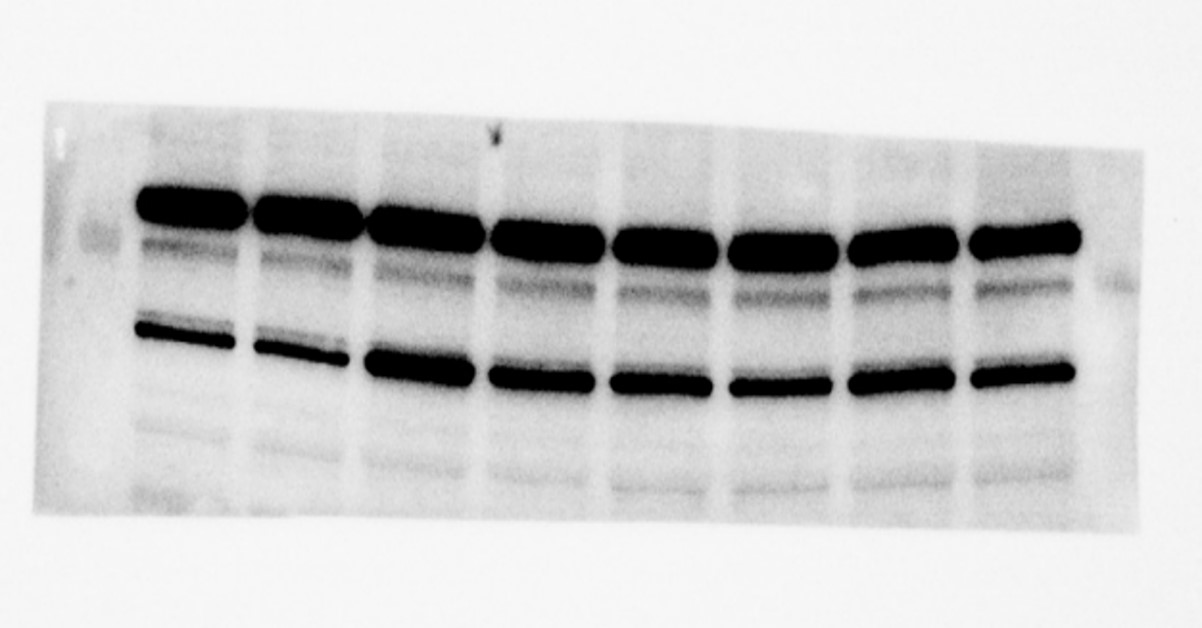

Supplement: Figure 5—source data 3. [file elife-83345-fig5-data3.zip › Source data 3 for Figure 5.jpg]

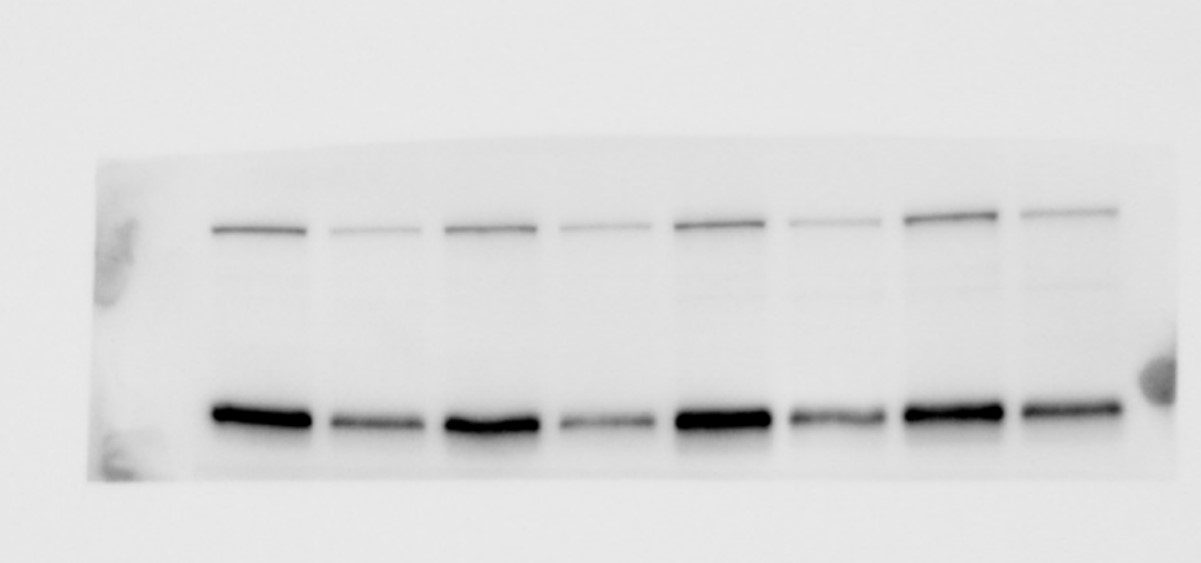

Supplement: Figure 5—source data 4. [file elife-83345-fig5-data4.zip › Source data 4 for Figure 5.jpg]

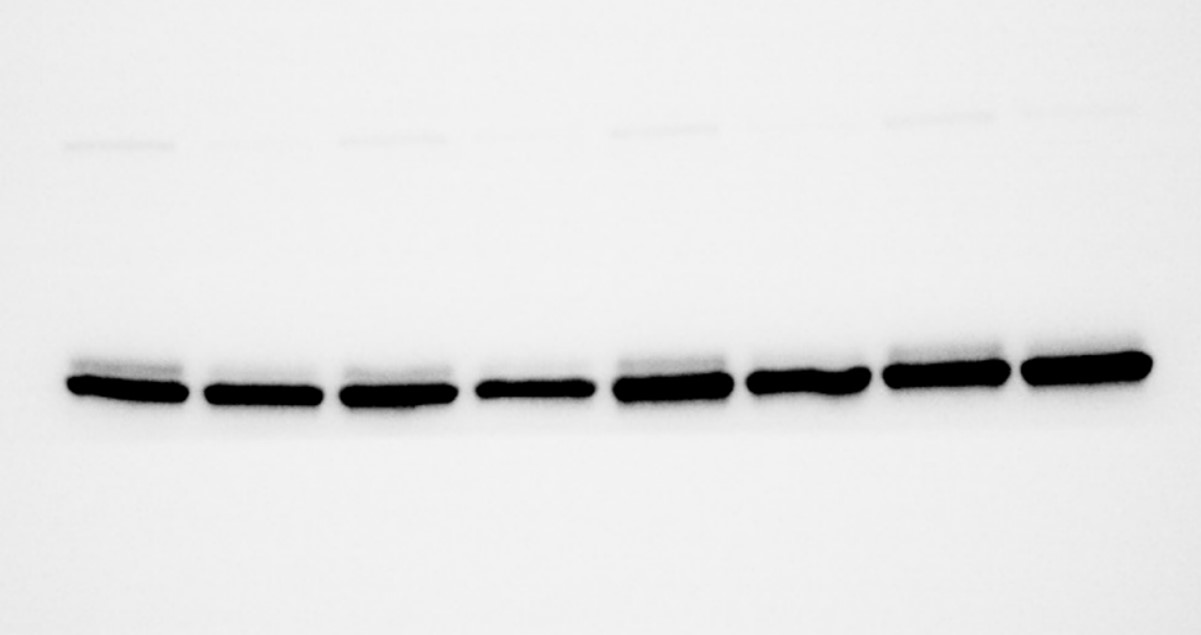

Supplement: Figure 5—source data 5. [file elife-83345-fig5-data5.zip › Source data 5 for Figure 5.jpg]

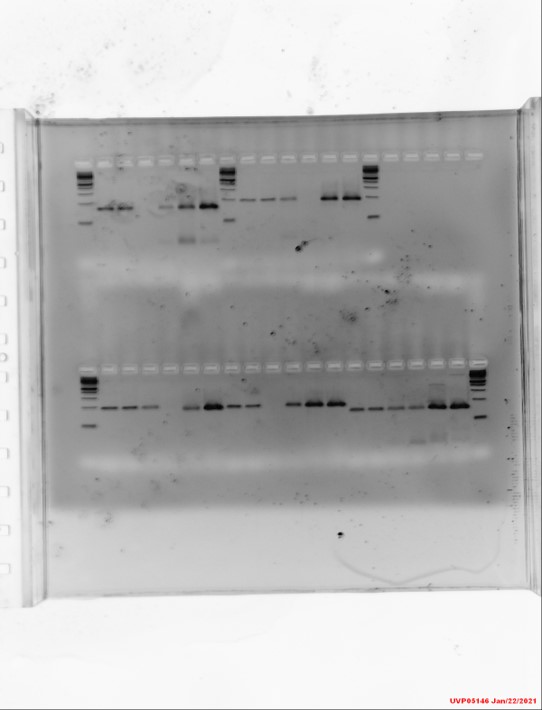

Supplement: Figure 5—figure supplement 1—source data 2. [file elife-83345-fig5-figsupp1-data2.zip › Source data 2 for Figure 5-figure supplement.jpg]

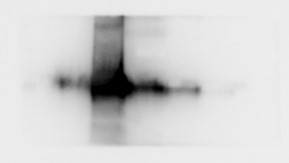

Supplement: Figure 6—source data 2. [file elife-83345-fig6-data2.zip › Source data 2 for Figure 6.jpg]

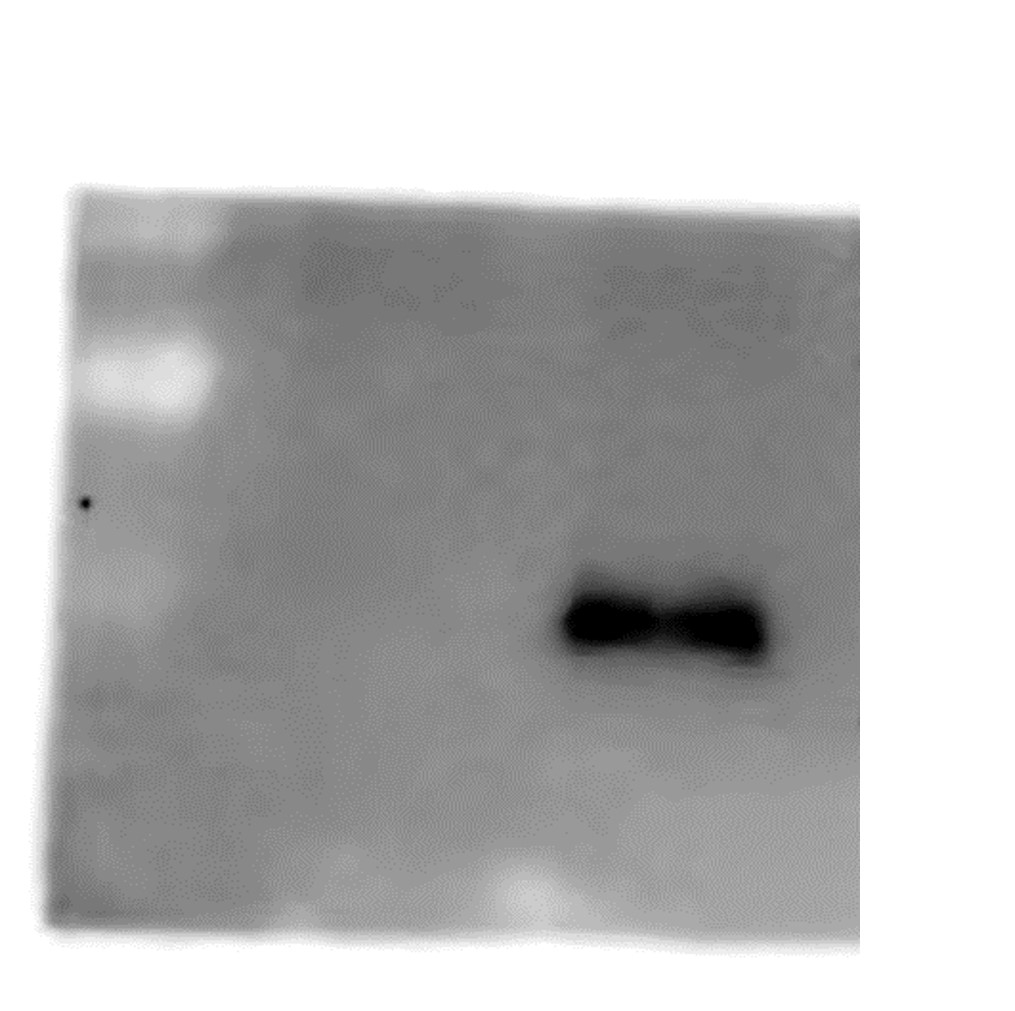

Supplement: Figure 6—source data 3. [file elife-83345-fig6-data3.zip › Source data 3 for Figure 6.jpg]

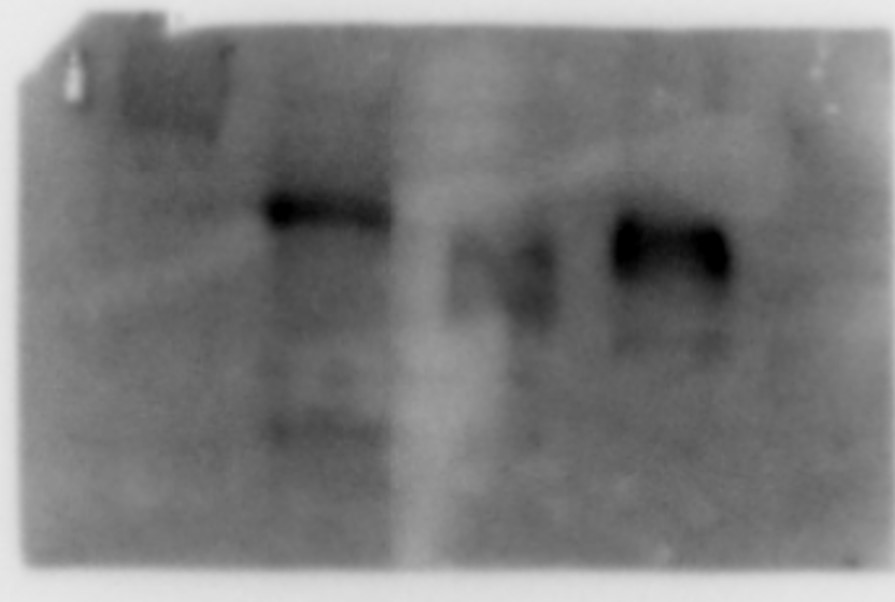

Supplement: Figure 6—source data 4. [file elife-83345-fig6-data4.zip › Source data 4 for Figure 6.jpg]

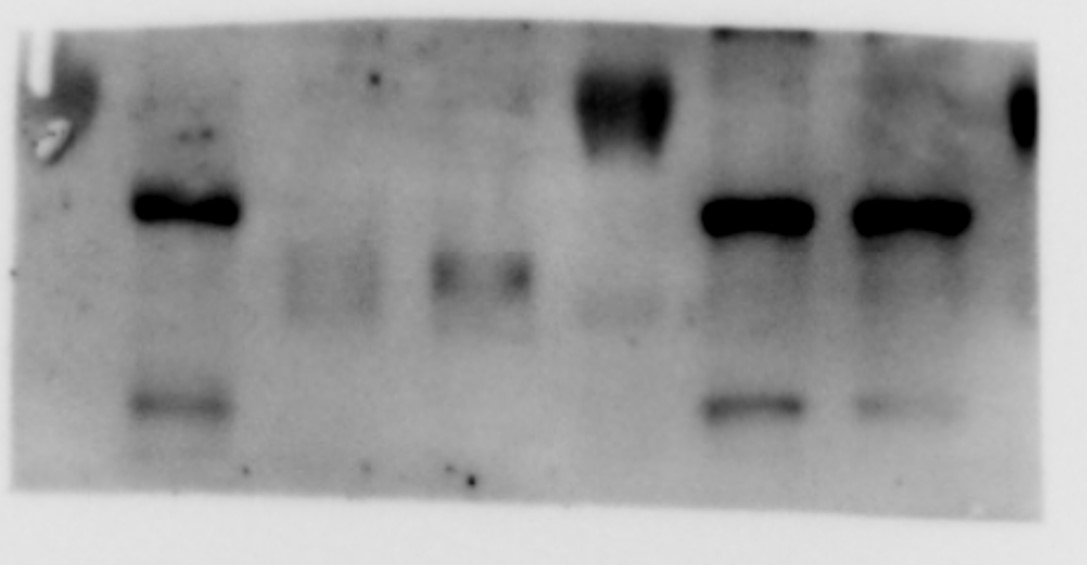

Supplement: Figure 6—source data 5. [file elife-83345-fig6-data5.zip › Source data 5 for Figure 6.jpg]

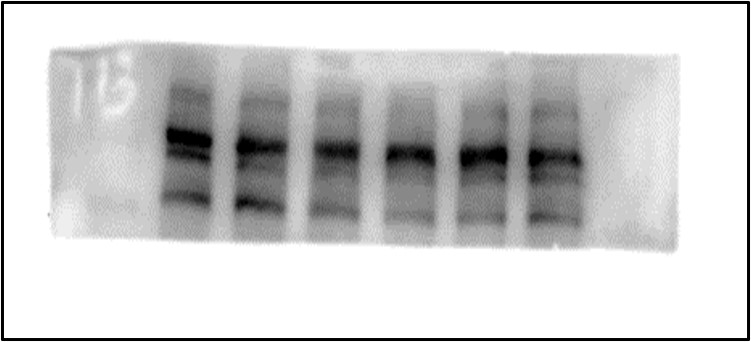

Supplement: Figure 6—source data 7. [file elife-83345-fig6-data7.zip › Source data 7 for Figure 6.jpg]

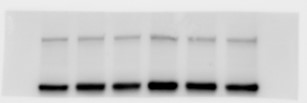

Supplement: Figure 6—source data 8. [file elife-83345-fig6-data8.zip › Source data 8 for Figure 6.jpg]

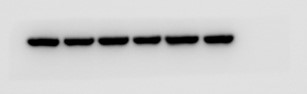

Supplement: Figure 6—source data 9. [file elife-83345-fig6-data9.zip › Source data 9 for Figure 6.jpg]

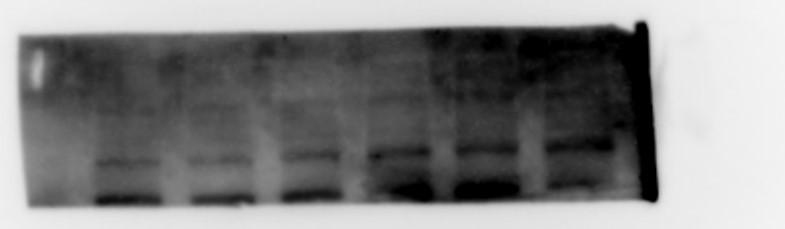

Supplement: Figure 6—source data 10. [file elife-83345-fig6-data10.zip › Source data 10 for Figure 6.jpg]

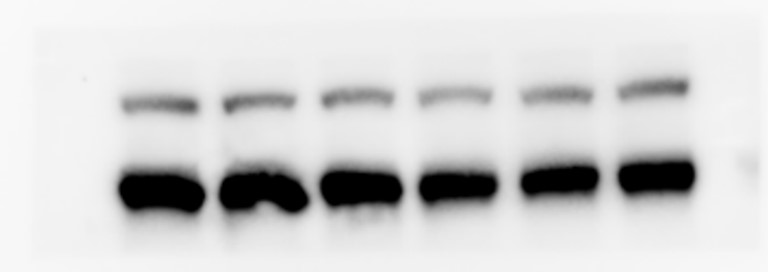

Supplement: Figure 6—source data 11. [file elife-83345-fig6-data11.zip › Source data 11 for Figure 6.jpg]

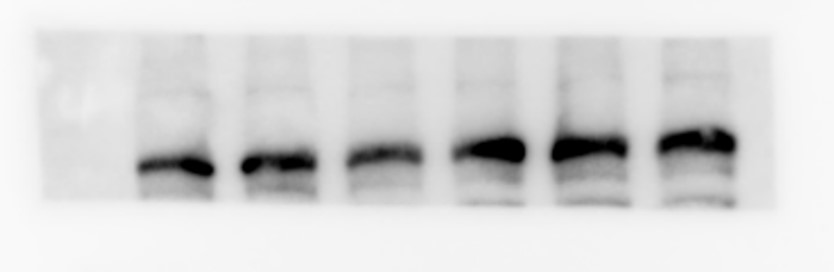

Supplement: Figure 6—source data 12. [file elife-83345-fig6-data12.zip › Source data 12 for Figure 6.jpg]

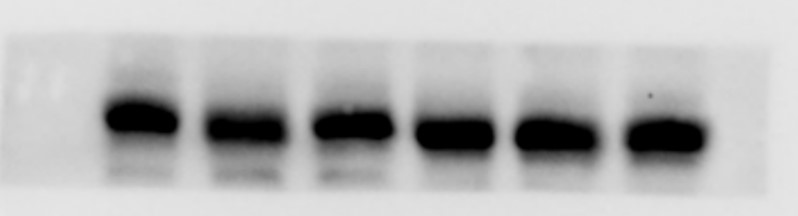

Supplement: Figure 6—source data 13. [file elife-83345-fig6-data13.zip › Source data 13 for Figure 6.jpg]

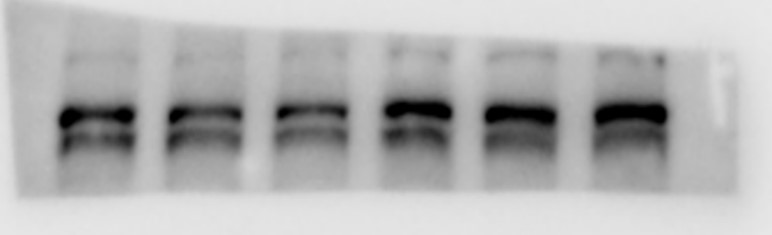

Supplement: Figure 6—source data 14. [file elife-83345-fig6-data14.zip › Source data 14 for Figure 6.jpg]

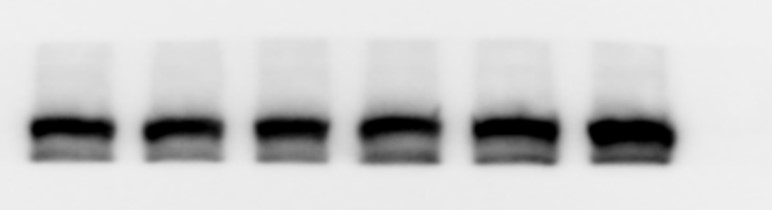

Supplement: Figure 6—source data 15. [file elife-83345-fig6-data15.zip › Source data 15 for Figure 6.jpg]

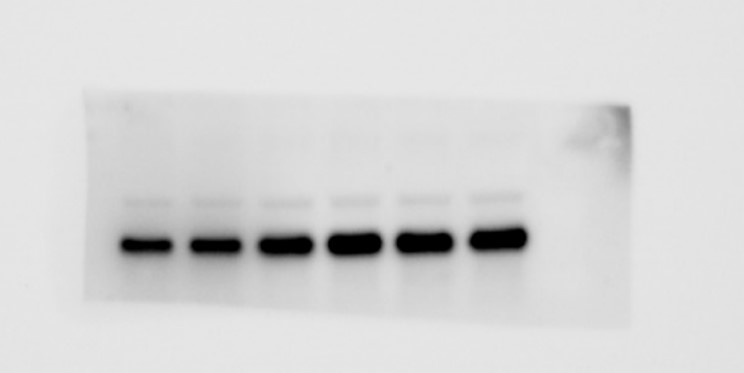

Supplement: Figure 7—source data 2. [file elife-83345-fig7-data2.zip › Source data 2 for Figure 7.jpg]

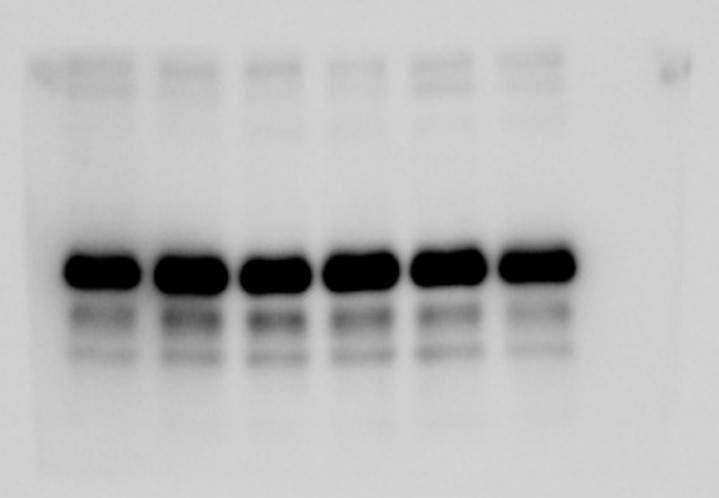

Supplement: Figure 7—source data 3. [file elife-83345-fig7-data3.zip › Source data 3 for Figure 7.jpg]

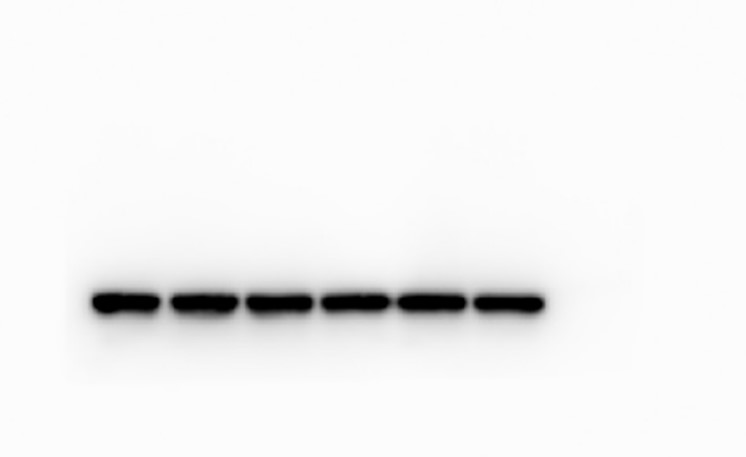

Supplement: Figure 7—source data 4. [file elife-83345-fig7-data4.zip › Source data 4 for Figure 7.jpg]

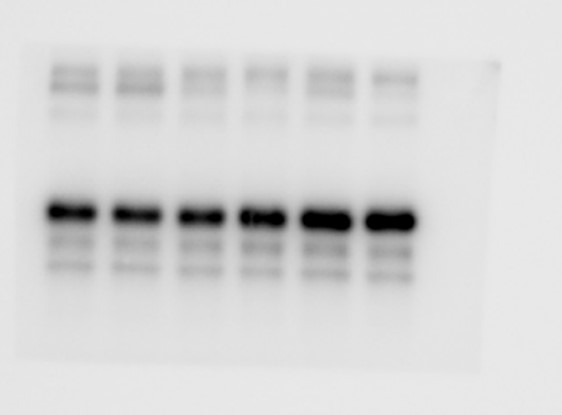

Supplement: Figure 7—source data 5. [file elife-83345-fig7-data5.zip › Source data 5 for Figure 7.jpg]

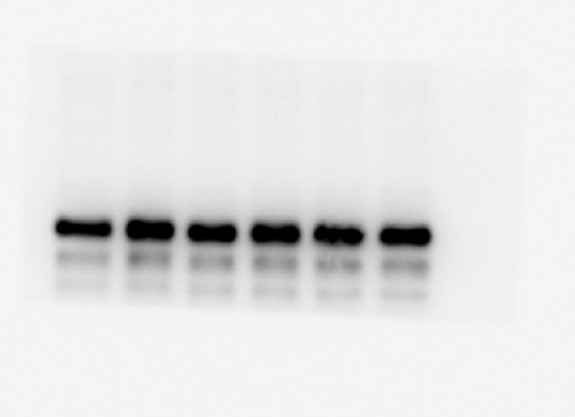

Supplement: Figure 7—source data 6. [file elife-83345-fig7-data6.zip › Source data 6 for Figure 7.jpg]

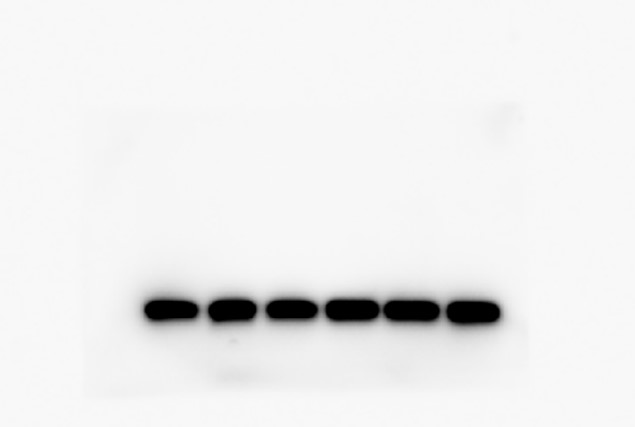

Supplement: Figure 7—source data 7. [file elife-83345-fig7-data7.zip › Source data 7 for Figure 7.jpg]
